# Serum miRNA-186-3P and miRNA-382-3P constitute a novel Diagnostic miRNA signature for palindromic rheumatism

**DOI:** 10.3389/fimmu.2025.1569846

**Published:** 2025-03-24

**Authors:** Fangfang Yuan, Zefu Weng, Qiong Yang, Jing Luo, Lina Ying, Haiyan Huang, Xin Zhang, Yahui Chen, Jixia Lin, Junhong He

**Affiliations:** ^1^ Department of Rheumatism and Immunology, Ningbo No. 6 Hospital, Ningbo, China; ^2^ Department of Orthopedics, Qianhu Hospital, Yinzhou District Dongqianhu Town Community Health Service Center, Ningbo, China; ^3^ Department of Pharmacy, Ningbo No. 6 Hospital, Ningbo, China; ^4^ Department of Clinical Laboratory, Ningbo No.6 Hospital, Ningbo, China

**Keywords:** palindromic rheumatism, serum miRNA-186-3P, serum miRNA-382-3P, miRNA signature, pathogenesis

## Abstract

**Background:**

Palindromic rheumatism (PR) is a unique disease characterized by the intermittent inflammation of different joints that may progress to a variety of immune-related diseases. Unclear diagnostic criteria have limited the research on its pathogenesis and treatment options. Recently, microRNAs (miRNAs) have been used in the diagnosis of various diseases; however, the role of miRNAs in PR diagnosis remains unexplored. Using next-generation high-throughput sequencing (NGS), this study aimed to screen miRNAs specifically expressed in the serum of patients with PR to construct a miRNA signature and verify its diagnostic efficacy.

**Methods:**

Patients with PR (N=4), patients with rheumatoid arthritis (RA; N=3), and healthy individuals (Con; N=3) were included in an exploration cohort. Differentially expressed miRNAs were screened using NGS to construct a miRNA signature, and bioinformatics tools were used to perform target gene enrichment analysis of the top 25 differentially expressed miRNAs, both upregulated and downregulated. RT-qPCR was used to verify the differential expression of the miRNA signature in three validation cohorts of patients with PR (N=27) and RA (N=30), and healthy individuals (N=31). The efficiency of the miRNA signature was evaluated using receiver operator characteristic (ROC) curves, an analytical method that assesses diagnostic accuracy.

**Results:**

A total of 130 miRNAs were differentially expressed in the PR exploration cohort, including 35 upregulated and 95 downregulated compared to levels in the RA and healthy cohorts. miRNA-186-3p showed the largest upregulated difference and miRNA-382-3p the largest downregulated difference; these were selected to construct the miRNA signature. In the ROC curve of the validation cohort, the PR miRNA signature produced an area under the ROC curve (AUC) of 0.980 (95% CI 0.942–1.000) when distinguishing from healthy individuals and of 0.906 (95% CI 0.830–0.983) when distinguishing from RA patients. However, miRNA-186-3p and miRNA-382-3p levels were not associated with disease activity in patients with PR.

**Conclusion:**

A miRNA signature comprising miRNA-186-3p and miRNA-382-3p can effectively diagnose and differentiate PR from RA. This study provides a basis for the creation of a clinical miRNA signature for the diagnosis of PR.

## 1 Introduction

Palindromic rheumatism (PR) is a clinical syndrome that involves pain and swelling in and around a single joint during an attack. It is characterized by variable joint locations and times of attack. After the symptoms subside, the patient has no residual damage and cannot be distinguished from healthy individuals (1). Early studies have found that PR has serological and immunogenetic characteristics similar to those of rheumatoid arthritis (RA) and a high incidence of progression to RA (2, 3). Therefore, PR is considered a relapsing–remitting form of RA. However, subsequent studies have not supported this conclusion. PR may present with extracapsular inflammation, which differs from RA when imaged using ultrasonography (3). The proportion of patients with PR that progress to RA ranged from 10–67% in different studies (4). PR also progresses to a wider range of rheumatic diseases than previously known, such as ankylosing spondylitis (AS) and systemic lupus erythematosus (SLE), among others (5, 6). Anti-RA drugs cannot be used directly to treat PR (7). These results indicate that PR cannot be regarded as a precursor to RA. Unfortunately, the lack of clear diagnostic criteria has limited the research on the pathogenesis and characteristics of PR. Different inclusion criteria have led to differences between related studies. Currently, the diagnosis of PR is still based on the experience of clinicians, requiring patients to visit the doctor multiple times, and PR continues to be easily misdiagnosed as RA.

MicroRNAs (miRNAs) are single-stranded non-coding RNAs that participate in the regulation of various biological pathways by regulating messenger-RNAs (mRNAs). miRNAs are associated with the secretion of pro-inflammatory cytokines and the promotion of inflammatory signaling pathways in various immune diseases; thus, they have the potential to be valuable therapeutic targets (8, 9). Importantly, due to their specificity and stability within body fluids, changes in miRNA expression in disease states can serve as biomarkers for diagnosis. The detection of a combination of several miRNAs used as a set of biomarkers is called a miRNA signature. Compared with a single miRNA biomarker, a miRNA signature is more reliable and likely to fully reflect the complexity of disease phenotypes (10). However, to date, no studies have been conducted on the role of miRNAs in PR.

Next-generation sequencing (NGS) techniques based on circular arrays are widely used in biomedical research. Among them, RNA sequencing (RNA-seq) reveals not only the differential expression of miRNAs in autoimmune diseases but also new miRNAs; consequently, it is an important tool for studying the pathogenesis of diseases (11). This study aimed to screen differentially expressed miRNAs in PR samples and compare them to those in RA and healthy individual samples using next-generation high-throughput RNA sequencing to construct a miRNA signature for PR diagnosis and verify its efficacy. Additionally, bioinformatics tools were used to perform target gene enrichment analysis on differentially expressed miRNAs, offering new insights into defining PR diagnostic criteria and studying its pathogenesis.

## 2 Materials and methods

### 2.1 Patients and serum sample collection for RNA studies

Serum samples from patients diagnosed with PR or RA were analyzed in this study. The ages of the patients ranged from 18–75 years. RA patients were diagnosed according to the 2010 classification criteria for rheumatoid arthritis (12), and those with PR according to the Guerne-Weisman criteria published in 1992 (13). Patients with PR in remission or flare-ups were included. Healthy individuals were volunteers from the physical examination center who had provided serum samples and had no related immune diseases. All patients and volunteers provided informed consent. Serum samples were collected in disposable human venous blood pressure collection tubes (Shandong Hongyu Medical Technology Group Co., Ltd., Weihei, China), according to standard procedures. After complete coagulation, the blood was centrifuged at 1800–2000 × g for 10–15 min, and whole blood was separated into serum and cellular components within 2 h of collection. Serum samples were stored at -80°C for subsequent analysis.

### 2.2 RNA extraction

The samples were removed from -80°C, thawed, and centrifuged, and the supernatant was collected. TRIzol LS reagent (Invitrogen Life Technologies, Carlsbad, CA, USA) was added to dissociate the nucleic acid–protein complex. Chloroform was then added to the extract, the resulting colorless aqueous phase was removed, and isopropanol was added to precipitate the RNA. Finally, ethanol was used to wash the RNA precipitate, the ethanol was removed, and RNase-free water was added to obtain the RNA solution, which was stored at -70°C. RNA purification and quantification were examined by spectrophotometry (NanoDrop ND 2000; Thermo Fisher Scientific, Waltham, MA, USA).

### 2.3 miRNA library preparation and sequencing

Total RNA from each sample was used to prepare the miRNA sequencing libraries. An NEBNext Multiplex Small RNA Library Prep Set for Illumina (New England Biolabs, Ipswich, MA, USA) was used according to the manufacturer’s instructions, and included the following steps: 1) 3’ adapter ligation; 2) 5’ adapter ligation; 3) cDNA synthesis; 4) PCR amplification; 5) selection of PCR amplified fragments of 135–155 bp (corresponding to small RNAs of 15–35 nt). The quality and concentration of the sequencing library were evaluated using an Agilent 2100 bioanalyzer (Agilent, Santa Clara, CA, USA).

The library was denatured into single-stranded DNA molecules using a TruSeq Rapid SR cluster kit (#GD-402-4001; Illumina, San Diego, California, USA), captured on an Illumina Flow cell (Illumina), amplified *in situ* into clusters, and sequenced on an Illumina sequencer (Illumina) according to the sequencing instructions. The sequencer generated raw sequencing data in the FASTQ format. The raw sequence of each sample was evaluated and found to have a sequencing quality score of Q30. The sequencing of miRNA in this study was completed by Aksomics Co., Ltd., Shanghai, China.

The normalization method for miRNA sequencing was counts per million (CPM). The process of miRNA expression quantification was as follows (1): the raw read count data were normalized and the CPM value of each gene calculated (2); log2 transformations were performed based on CPM values (3); mirdeep2 version 0.0.8 software was used to map the results to the known genome and perform statistical analysis to quantitatively measure miRNA expression. Those miRNAs with mean CPM ≥1 were considered to be expressed in that group and were statistically analyzed. The specific calculation formula for CPM is as follows:


$$\mathrm{CPM}=\frac{\text{C}\times 10^{6}}{\text{N}}$$

where C is the number of reads mapped to a gene and N is the total number of reads mapped to all genes.

### 2.4 Functional and pathway enrichment analysis

Gene ontology analysis was performed on the differentially expressed miRNAs, according to the molecular function (MF), biological process (BP), and cellular component (CC) domains. Pathway enrichment analysis was performed based on the latest biological pathway classification catalog in the Kyoto Encyclopedia of Genes and Genomes (KEGG) database. All enrichment analyses were performed using R version 3.4.1 software. Differences with P < 0.05 were considered statistically significant. The TargetScan and miRDB databases were used to predict miRNA target genes.

### 2.5 Quantitative polymerase chain reaction

The screened miRNA signature was reverse-transcribed and synthesized into cDNA using the Gene Amp PCR System 9700 (Applied Biosystems, Foster City, CA, USA). The sample volume used during quantitative PCR was 2 µL. However, due to the influence of RNA concentration quantification and reverse-transcription efficiency errors, the cDNA content of each 2 µL sample varied considerably. To correct for these differences, we used hsa-miR-423-5p content as an internal reference because its expression level among different samples was constant. PCR was performed using the QuantStudio5 Real-time PCR System (Applied Biosystems) with the following cycling conditions: initial denaturation at 95°C for 10 min, followed by 40 cycles of denaturation at 95°C for 10 s, and annealing/elongation at 60°C for 60 s. To establish the melting curve for the PCR product, the amplification was followed by heating to 95°C for 10 s, cooling to 60°C for 60 s, heating to 95°C for 15 s, and finally by gradual heating from 60°C to 99°C at a ramp rate of 0.075°C/s (automatically performed by the instrument). Data were analyzed using the 2^-△△CT^ method.

### 2.6 Statistical analysis

Data were processed using SPSS version 16.0 (IBM, Armonk, NY, USA) and GraphPad Prism 9 (GraphPad Software, La Jolla, CA, USA), and statistical significance was set at P < 0.05. Normally distributed and non-normal quantitative data were expressed as mean ± SD and median (interquartile range), respectively. Differential miRNA expression between groups was analyzed using edgeR (3.18.1) software. The threshold for differential expression was set to a 1.5-fold difference, P-value < 0.05, and CPM mean within the group >1. The statistical correction method for multiple comparisons was false discovery rate (FDR), calculated using the Benjamini–Hochberg (BH) algorithm of the p.adjust function in R 4.2.2 software. Cluster analysis was performed using the CPM values ​​of the significantly expressed miRNAs obtained from comparisons between groups. Principal component analysis (PCA) was performed using R version 4.2.2. R 4.2.2 was also used to perform stratified k-fold cross validation, plot receiver operating characteristic (ROC) curves, and calculate the area under the ROC curve (AUC). The k value was selected as 5 to balance the size of the training set and the reliability of the test set. In each iteration, the training and validations set were completely independent, with no sample overlap. Only the training set data was used to calculate the parameters and apply them to the validation set. The training and validation sets were strictly isolated.

## 3 Results

Between September 2023 and 2024, 95 participants were enrolled in this study, including 31 patients with PR, 33 with RA, and 31 healthy individuals. The average age of the patients with PR was 39.97 ± 12.15 years, and the male to female ratio was 2.44:1; both were significantly different from those of RA patients (P=0.0013 and P<0.0001, respectively). The main occupation of the patients with PR was office clerk (67.7%), while that of those with RA was farmer (51.5%). Most patients with PR had a high school education or above (71.0%), whereas only a small number of those with RA had completed high school (21.2%). The treatment duration was significantly shorter in patients with PR than in those with RA (0.5 vs 4.0 years, P<0.0001). The distribution of involved joints was similar in the PR and RA groups, mainly including hand, knee, wrist, and elbow joints, followed by shoulder, feet, and ankle joints. Of the patients with PR, 54.8% had no comorbidities, whereas 54.5% of those with RA had three or more chronic diseases. Compared to RA patients, those with PR had significantly lower proportions of positive rheumatoid factor (RF) and anti-cyclic citrullinated peptide (anti-CCP) antibodies (16.1%, P<0.0001; 16.1%, P<0.0001; respectively). Similarly, the erythrocyte sedimentation rate (ESR) and C-reactive protein (CRP) expression levels were also lower in patients with PR than in those with RA (**Table 1**).

**Table 1:** Clinical characteristics of participants.

	PR (N=31)	RA (N=33)	Con (N=31)	P
**Age(± SD)**	37.97 (12.15)	47.70 (12.7)	37.74 (11.33)	0.0013
Sex (%)				
Male	22 (71.0%)	9 (27.3%)	22 (71.0%)	<0.0001
Female	9 (29.0%)	24 (72.4%)	9 (29.0%)	
Occupation				
Worker	4 (12.9%)	9 (27.3%)	N/A	<0.0001
Farmer	2 (6.5%)	17 (51.5%)	N/A	
Office clerk	21 (67.7%)	6 (18.2%)	N/A	
Freelance work	0 (0.0%)	1 (3.0%)	N/A	
Unemployed	4 (12.9%)	0 (0%)	N/A	
Education Background (%)				
None	0 (0.0%)	4 (12.1%)	N/A	<0.0001
Below high school	9 (29.0%)	22 (66.7%)	N/A	
High school and above	22 (71.0%)	7 (21.2%)	N/A	
**Course of disease (IQR)**	2.0 (0.67-5.00)	4.0 (2.00-6.50)	N/A	0.0690
**Course of treatment (IQR)**	0.5 (0.25-1.00)	4.0 (2.00-5.00)	N/A	<0.0001
**Pain Score (IQR)**	3.0 (2.00-3.00)	3.0 (3.00-3.00)	N/A	0.9007
**Frequency of Flares (IQR)**	3.0 (1.00-3.00)	N/A	N/A	N/A
**Involved joints (%)**			N/A	N/A
Hand	19 (61.29%)	31 (93.94%)		
Wrist	13 (41.93%)	25 (75.76%)		
Elbow	10 (32.26%)	24 (72.73%)		
Shoulder	6 (19.35%)	15 (45.45%)		
Knee	18 (58.06%)	27 (81.82%)		
Hip	3 (9.68%)	3 (9.68%)		
Ankle	6 (19.35%)	14 (42.42%)		
Foot	11 (35.48%)	12 (36.36%)		
Complicating Disease (%)				
None	17 (54.8%)	11 (33.3%)	N/A	<0.005
Single disease	9 (29.2%)	4 (12.1%)		
Multiple diseases	5 (16.1%)	18 (54.5%)		
Drug (%)				
Single drug	14 (45.2%)	3 (9.1%)	N/A	<0.001
Multiple drugs	17 (54.8%)	30 (90.9%)		
**RF (IQR)**	4.8 (2.40-7.20)	83.1 (39.20-147.0)	N/A	<0.0001
Positive	5 (16.1%)	28 (84.8%)		<0.0001
Negative	26 (83.9%)	5 (15.2%)		
**Anti-CCP (IQR)**	7.0 (7.00-8.00)	301.0 (151.3-489.1)	N/A	<0.0001
Positive	5 (16.1%)	28 (84.8%)		<0.0001
Negative	26 (83.9%)	5 (15.2%)		
**ESR (IQR)**	22.0 (9.75-49.00)	45.0 (29.50-83.00)	N/A	0.0082
**CRP (IQR)**	4.9 (1.30-9.10)	12.4 (7.50-31.25)	N/A	0.0017
**DAS28**	N/A	5.5 (5.15-5.85)	N/A	N/A

PR, palindromic rheumatism; RA, rheumatoid arthritis; Con, health controls; IQR, interquartile range; RF, rheumatoid factor; Anti-CCP, anti-cyclic citrullinated peptide; ESR, erythrocyte sedimentation rate; CRP, C-reactive protein; DAS28, Disease Activity Score 28.

### 3.1 Differentially expressed miRNAs in patients with PR

**Figure 1:**
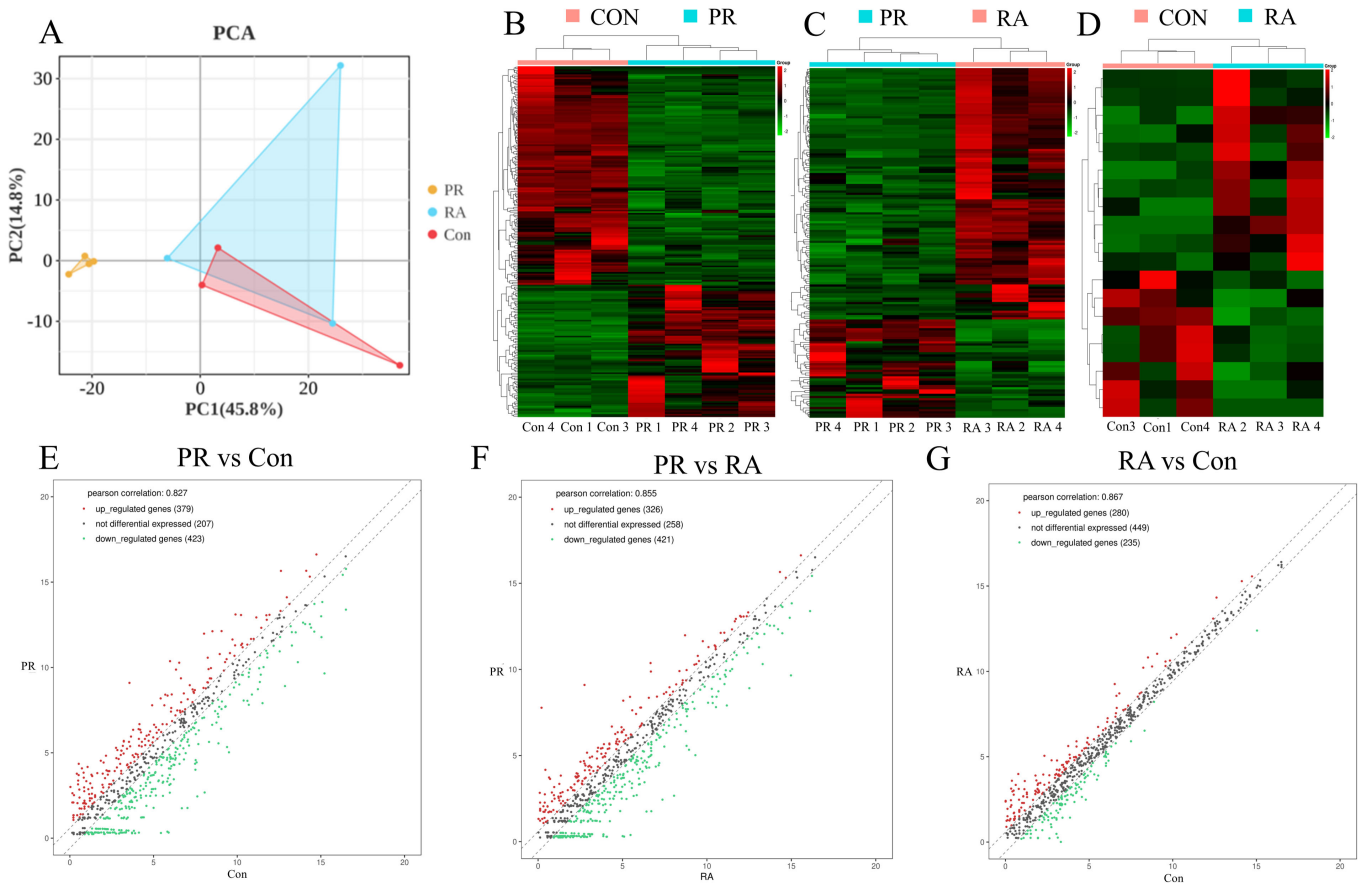
Principal component analysis (PCA) and cluster analysis of miRNA expression in patients with palindromic rheumatism (PR) rheumatoid arthritis (RA), and healthy individuals (Con). **(A)** Global PCA of miRNA expression between PR (yellow), RA (blue), and healthy control (red) samples; **(B–D)** supervised hierarchical clustering analysis of miRNA expression profiles compared among groups; **(E–G)** scatter plots of miRNA expression profiles compared between groups.

To screen for differentially expressed miRNAs among patients with PR and RA and healthy individuals, an exploratory cohort was formed consisting of four patients with PR (N=4), three with RA (N=3), and three healthy individuals (N=3) (**Supplementary Table S1**). Expressed miRNAs were identified in the three groups and analyzed using PCA and hierarchical clustering. In the PCA, patients with PR were clearly distinguishable from those with RA and healthy individuals, whereas RA patients were indistinguishable from healthy individuals (**Figure 1A**). Heat maps and scatter plots also showed that the differential miRNA signature of patients with PR was distinct from that of those with RA and healthy individuals (**Figures 1B–G**). The volcano plots constructed for comparison between the groups also supported this finding (**Supplementary Figure S1**).

### 3.2 Functional annotation and pathway enrichment analysis

**Figure 2:**
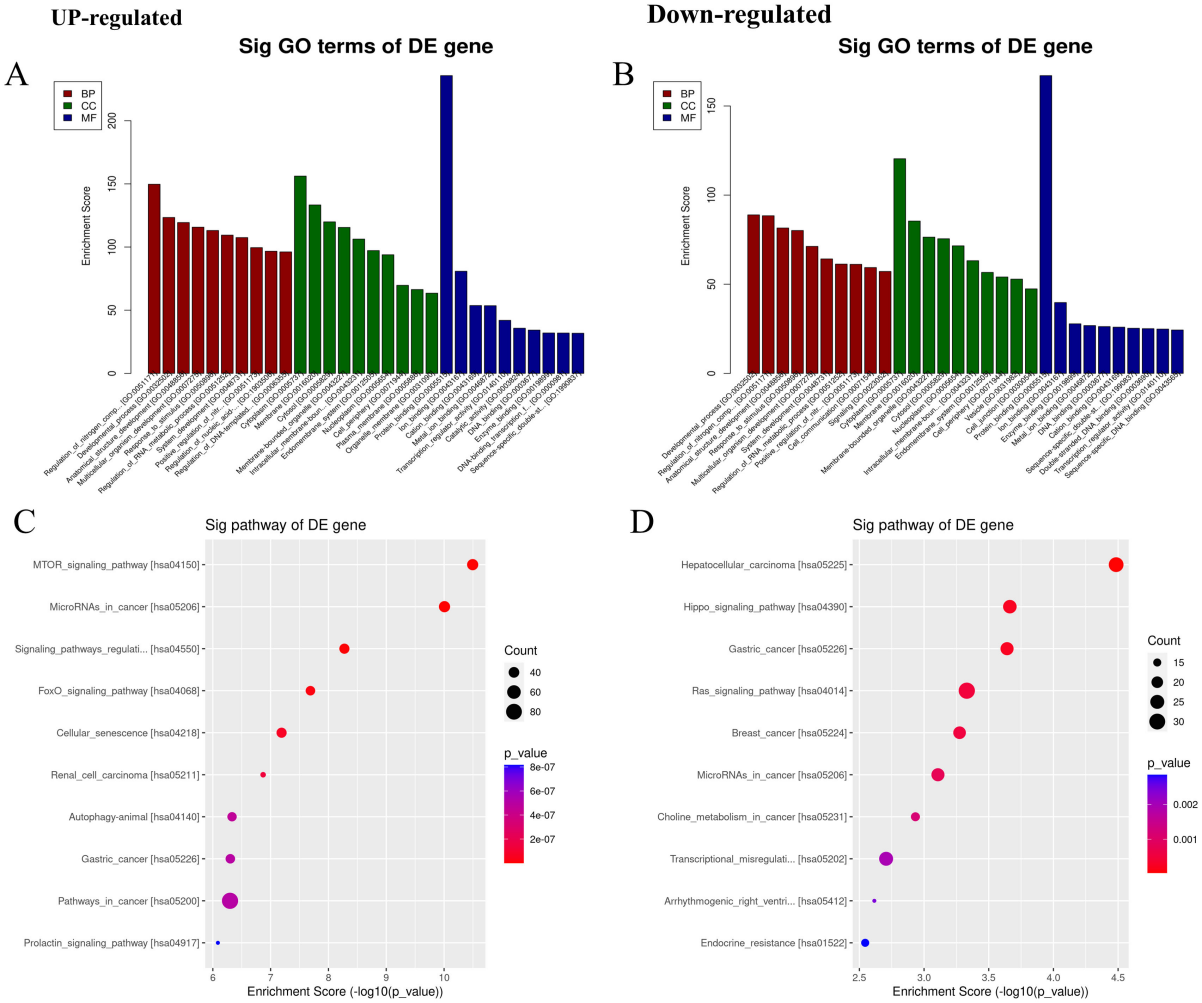
Gene ontology (GO) analysis and Kyoto Encyclopedia of genes and genomes (KEGG) pathway enrichment analysis. **(A, B)** The top 10 GO entries with the most significant differences in the GO analysis (biological process (BP), cellular component (CC), and molecular function (MF)) results of upregulated and downregulated miRNAs. The ordinate is the P-value (-log10 transformation). **(C, D)** The top 10 items with the most significant differences in the results of KEGG pathway enrichment analysis between upregulated and downregulated miRNAs. The ordinate is the P-value (-log10 transformation).

A total of 130 differentially expressed miRNAs were found in the PR exploration cohort, 35 upregulated and 95 downregulated compared to levels in both the RA and healthy cohorts. The top 25 differentially expressed miRNAs were selected for target gene enrichment analysis (**Supplementary Table S2**). Gene ontology analysis showed that the upregulated miRNAs were mainly enriched in protein binding (MF domain), regulation of nitrogen compound metabolism (BP domain), and cytoplasm-related processes (CC domain), whereas the downregulated miRNAs were mainly enriched in protein binding (MF), developmental processes (BP), and cytoplasm-related processes (CC; **Figures 2A, B**). In KEGG pathway enrichment analysis, upregulated miRNAs were mainly enriched in the mTOR signaling pathway and miRNAs in cancer, whereas downregulated miRNAs were mainly enriched in hepatocellular carcinoma (**Figures 2C, D**). Pathway analysis revealed that the upregulated miRNAs may downregulate miRNAs in the mTOR signaling pathway and primarily affect the MAPK signaling pathway. The downregulated miRNAs may affect the Wnt and PI3K–Akt signaling pathways in hepatocellular carcinoma (**Supplementary Figure S2**).

### 3.3 Construction and validation of the miRNA signature for PR

The top three upregulated and downregulated miRNAs were evaluated to construct the PR miRNA signature. miR-186-3p and miR-382-3p were significantly upregulated and downregulated, respectively, in patients with PR compared to levels in the RA and healthy cohorts (**Supplementary Figure S3**). Using miR-423-5p expression as an internal reference (14), the expression levels of miR-186-3p and miR-382-3p were verified in the three sample groups (**Table 2**).

**Table 2:** Sequence and primers of miRNAs.

miRNA	Mature sequence 5′–3′	Bidirectional primer sequences
miR-382-3p	AAUCAUUCACGGACAACACUU	GSP:5′GGGCAATCATTCACGGACA3’ R:5’GTGCGTGTCGTGGAGTCG3’
miR-186-3p	GCCCAAAGGUGAAUUUUUUGGG	GSP:5′GGCAGCCCAAAGGTGAAT3′ R:5′GTGCGTGTCGTGGAGTCG3′

GSP, The specific primer corresponding to miRNA; R, the primer matching the RT primer.

**Figure 3:**
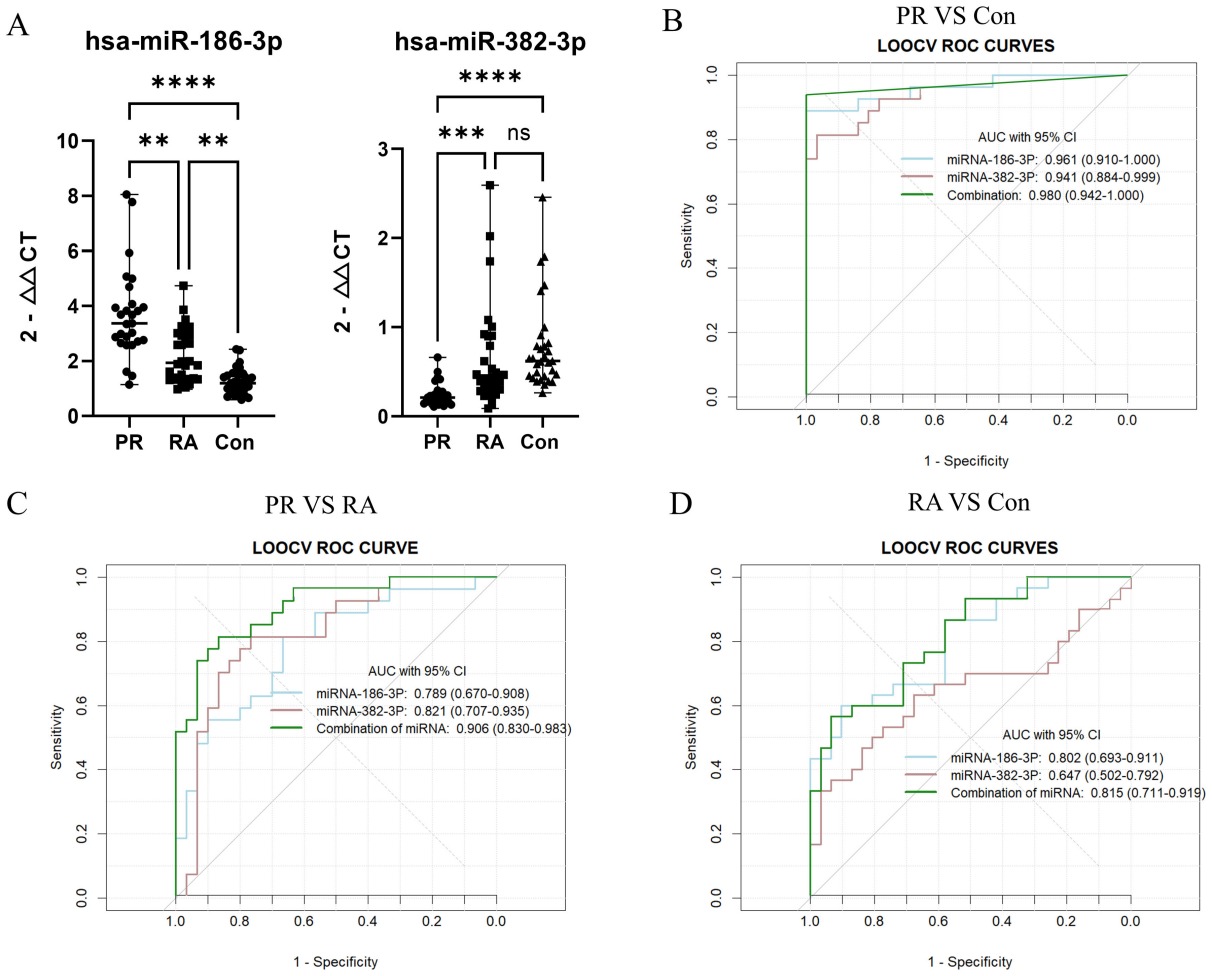
**(A)** The expression levels of miR-186-3p and miR-382-3p in the serum of each group; **(B)** ROC curve analysis of miRNA-186, miRNA-382, and the miRNA signature (palindromic rheumatism (PR) vs healthy individuals (Con)); **(C)** ROC curve analysis of miRNA-186, miRNA-382, and the miRNA signature (PR vs rheumatoid arthritis (RA)); **(D)** ROC curve analysis of miRNA-186, miRNA-382, and the miRNA signature (RA vs Con). “**” indicates that the data were statistically significant, P < 0.01; “***” indicates that the data were statistically significant, P < 0.001; “****” indicates that the data were statistically significant, P < 0.0001; “ns” indicates that the data were not statistically significant, P > 0.05.

**Figure 4:**
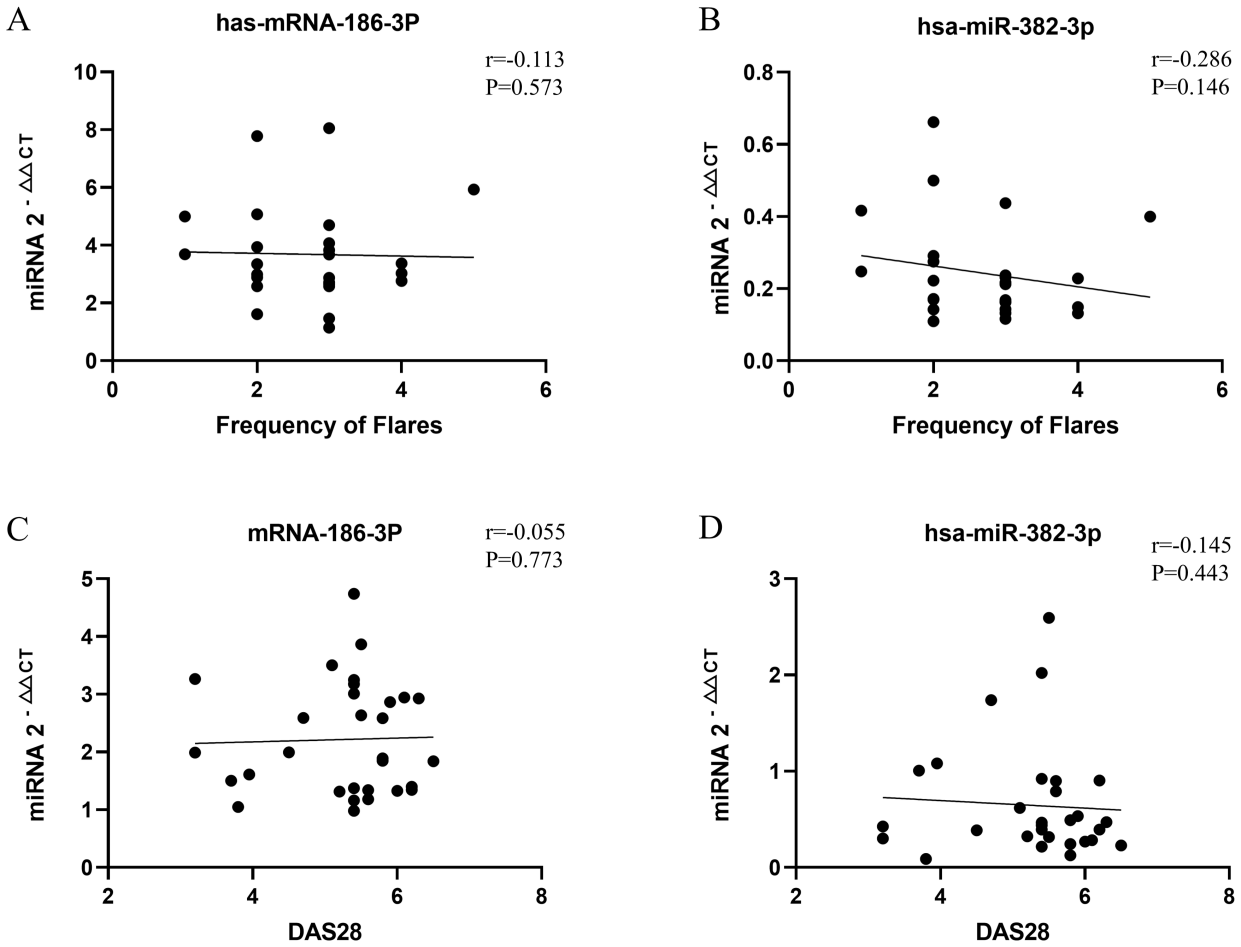
Correlation analysis between monthly attack frequency of patients with palindromic rheumatism (PR) and miR-186-3p **(A)** and miR-382-3p **(B)** expression; correlation analysis between disease activity score 28 (DAS28) of patients with rheumatoid arthritis (RA) and miR-186-3p **(C)** and miR-382-3p **(D)** expression.

miR-186-3p expression was significantly higher in the serum of patients with PR than in those with RA and healthy individuals, whereas miR-382-3p expression was significantly lower. miR-186-3p expression was significantly higher in RA patients than in healthy individuals, but miR-382-3p expression showed no significant difference (**Figure 3A**). In ROC curve analysis, the AUC values ​​of miR-186-3p and miR-382-3p for diagnosing PR were >0.9 compared with those for healthy individuals. The AUC value of the miRNA signature comprising miR-186-3p and miR-382-3p reached 0.980 (95% CI 0.942–1.000; **Figure 3B**). When used to distinguish patients with PR from those with RA, the AUC values of miR-186-3p and miR-382-3p were 0.789 (95% CI 0.671–0.908) and 0.821 (95% CI 0.707–0.935), respectively, and the AUC value of the miRNA signature was 0.906 (95% CI 0.830–0.983) (**Figure 3C**). Convergence analysis of the training and validation curves showed no overfitting (**Supplementary Figure S4**). However, when comparing RA patients and healthy individuals, the AUC values of miR-382-3p and the miRNA signature decreased (**Figure 3D**). In addition, the disease activity of PR and RA showed no correlation with miR-186-3p or miR-382-3p levels (**Figure 4**).

## 4 Discussion

As there are currently no widely accepted diagnostic criteria for PR nor a clear pathogenesis, this study screened differentially expressed miRNAs in serum samples from patients with PR, RA patients, and healthy individuals. We aimed to construct a miRNA signature for PR diagnosis. The average age of patients with PR in this study was lower than that of those with RA. In terms of occupation, the patients with PR were mainly office clerks, whereas those with RA were mainly farmers. All patients with PR were educated, and 71.0% had received a high school education or above. However, most RA patients (78.8%) lacked a high school education. Office clerks often have more regular working hours and sufficient time to visit doctors multiple times to diagnose PR. Patients with higher levels of education are generally more concerned about their health. This explains the high proportion of educated patients with PR. This also suggests that the prevalence of PR is higher than observed. Two previous studies found that 16–18% of patients diagnosed with RA had a history of PR (15, 16). Some patients with PR may have developed RA before being diagnosed with PR, especially those with low levels of education. The lack of specific diagnostic criteria has led to delays in patient treatment and variability in the patients included in different studies. Therefore, new diagnostic markers for PR are urgently needed.

In this study, we used NGS technology and found that >20% and >15% of detectable miRNAs in the serum samples of patients with PR were significantly different from those in healthy individuals and RA patients, respectively. Notably, less than 2% of miRNAs were differentially expressed between the RA and healthy cohorts. This indicates that patients with PR can be distinguished from those with RA and healthy individuals by detecting serum miRNAs. We selected the most significantly upregulated and downregulated miRNAs in PR and constructed a miRNA signature to distinguish patients with PR from those with RA and healthy individuals.

miR-186-3p was the most upregulated, and subsequent q-PCR revealed that its expression was significantly higher in the serum of patients with PR than in those with RA and healthy individuals. Further, miR-186-3p expression was significantly higher in RA than in the healthy cohort. Most studies on miR-186-3p have focused on cancer. miR-186-3p levels are reduced in colorectal cancer tissues (17) and cervical cancer (18, 19). However, higher plasma levels of miR-186-3p were observed in renal cell carcinoma samples (20). In addition, miR-186-3p is a tumor suppressor in non-small cell lung cancer (NSCLC), which can directly target kinesin family member 3C (KIF3C) to inhibit its expression, thereby inhibiting the proliferation and metastasis of NSCLC cells (21). According to recent studies, miR-186-3p also affects important signaling pathways in autoimmune diseases. The mRNA and protein levels of MYC, CD47, and PD-L1 are significantly reduced by miR-186-3p (17). CD47 can effectively mediate the release of inflammatory cytokines such as IL-12, TNF-α, and interferon-γ, affecting the pathogenesis of osteoarthritis (OA) (22, 23). MYC is involved in heterotopic ossification in AS (24). Thus, the PD-1/PD-L1 pathway may also play an important role in the pathogenesis of RA (25). In addition, miR-186-3p can affect the PI3K/AKT/mTOR signaling pathway by inhibiting IGF1 and KIF3C (19). The mTOR signaling pathway plays a key role in the pro-inflammatory and anti-inflammatory responses of various autoimmune diseases, including RA, SLE, Sjögren’s syndrome, and spondyloarthropathies (26). Notably, in the pathway enrichment analysis in the present study, the upregulated miRNA target genes were mainly enriched in the mTOR signaling pathway. This suggests that the mTOR signaling pathway is involved in the pathogenesis of PR and may be regulated by miR-186-3p. The increased miR-186-3p expression may be due to negative feedback upregulation caused by the inflammatory response in RA. The reduced inflammatory response in patients with PR without attacks may be due to the strong inhibitory effects of miR-186-3p. We speculate that as PR progresses, miR-186-3p expression decreases owing to the weakening of its negative feedback regulatory ability, which is a possible mechanism for the gradual progression to persistent joint inflammation.

miR-382-3p was the most downregulated miRNA in patients with PR, and its expression was significantly lower in patients with PR than in those with RA and healthy individuals. Similar to miR-186-3p, miR-382-3p has been reported in cancer-related studies. In pancreatic cancer, ovarian cancer, and glioma, miR-382-3p is involved in the regulation of tumor cell proliferation and metastasis (27–29). miR-382-3p overexpression reduces the expression of pro-inflammatory cytokines and MMP-1/13 stimulated by interleukin-1 beta (IL-1β) and has a protective effect against the IL-1β-induced inflammatory response. The inhibitory effect of miR-382-3p on TLR4, MyD88, and NF-кB expression regulates inflammatory responses and progressive joint destruction in OA and RA (30, 31). In addition, miR-382-3p is enriched in endothelial progenitor cell-derived exosomes, which alleviated cecal ligation and puncture-induced organ damage and immunosuppression in septic mice by regulating the phosphorylation of NF-κB (32). Notably, NF-κB plays a crucial role in the inflammatory process of RA (33). Based on the above studies and the high-throughput sequencing results in the present study, we speculate that miR-382-3p plays a key role in PR pathogenesis. Lower miR-382-3p levels likely reduce the inhibitory effect on the inflammatory response and progressive joint destruction, which may play a key role in PR pathogenesis. miR-382-3p expression in RA patients was indistinguishable from that in healthy individuals, and miR-382-3p did not appear to play a significant role in RA pathogenesis. These results indicate that PR and RA are closely related but should not be considered the same disease.

In ROC analysis compared with healthy individuals, the AUC values ​​of miR-186-3p and miR-382-3p were >0.9, and the AUC value of the miRNA signature composed of the two was 0.980, indicating high diagnostic performance. Compared with RA, the AUC value of the miRNA signature was still >0.9; notable, when comparing the expression of miR-382-3p, PR and RA were distinguishable. miR-186-3p and miR-382-3p expression levels were unrelated to PR and RA disease activity, possibly because patients were not stratified according to disease activity in this study. miR-186-3p and miR-382-3p expression may represent a certain state in patients with PR, or may simply be an epiphenomenon of the disease. However, miRNA-186-3p and miRNA-382-3p may still have value as qualitative diagnostic markers. If they reflect early disease, their stable expression may indicate the upstream regulatory mechanism of PR pathophysiology and be unrelated to the fluctuation of clinical symptoms. Other regulatory molecules or proteins downstream of miR-186-3p and miR-382-3p may cause intermittent and migratory inflammatory attacks in patients with PR. If the differential expression is an epiphenomenon, it can still provide an auxiliary basis for PR diagnosis, and its being unrelated disease activity may improve the stability of detection. Future studies could further verify the diagnostic efficacy of these features and their associations with PR disease activity through more sophisticated grouping designs (such as acute vs stable phase samples) or longitudinal tracking.

This study had some limitations. First, the sample size was small due to the number of patients with PR included and funding limitations. Although measures were taken to reduce selection bias, the small exploratory cohort still increased the risk of false positives and might have missed potential biomarkers with small and medium effect sizes. Second, the single-center study design lacked external validation in an independent data set, and the results might not be applicable to all populations. We will conduct a multicenter study involving different regions and ethnicities to verify the miRNA signature in the future. Third, this study did not stratify patients by disease activity (e.g., attack/remission status, different drug treatment groups, symptom severity stratification, etc.), nor did it longitudinally track changes in miRNA expression in the same patient at different stages of the disease course. Therefore, capturing the potential association between miRNA and disease activity using the existing data is difficult. Fourth, owing to funding constraints, we only selected two miRNAs to construct the miRNA signature and for subsequent verification. The effects of the other differentially expressed miRNAs on PR diagnosis and disease activity have not yet been studied. Finally, this study did not verify the changes in the downstream signaling pathways and functional proteins of miR-186-3p and miR-382-3p in patients with PR.

## 5 Conclusions

This study is the first to investigate the differential expression of serum miRNAs in PR compared to that in RA and healthy individuals. Additionally, a miRNA signature was constructed that can effectively distinguish patients with PR from those with RA and healthy individuals. miR-186-3p and miR-382-3p expression may be a feature of patients with PR, independent of disease activity. The detection of serum miR-186-3p and miR-382-3p expression can aid in the diagnosis and initiation of treatment for patients with PR. In addition, the functional annotation and pathway enrichment analysis of differentially expressed miRNAs in patients with PR in this study indicated that the PR pathogenesis is closely related to but distinct from that of RA, providing new insights into the development and treatment of PR. This study lays the groundwork for research into other miRNAs in the context of PR and the development of a clinically viable diagnostic miRNA signature for the diagnosis of PR.

## Data Availability

The datasets presented in this study can be found in online repositories. The names of the repository/repositories and accession number(s) can be found below: https://www.ncbi.nlm.nih.gov/geo/, GSE288542.
