# Supplementary material for: Serum miRNA-186-3P and miRNA-382-3P constitute a novel Diagnostic miRNA signature for palindromic rheumatism
Source: Front Immunol. 2025 Mar 24;16:1569846. doi: 10.3389/fimmu.2025.1569846 (PMC11973322; doi:10.3389/fimmu.2025.1569846)
Supplement: Supplementary file 1 [file DataSheet1.docx]

**Table S1. Clinical and biological characteristics of the exploration cohorts**

|  | **PR**  **（N=4）** | **RA**  **（N=3）** | **Con**  **（N=3）** |
| --- | --- | --- | --- |
| **Age(±SD)** | 44.00（8.37） | 46.67（11.15） | 49.75（4.27） |
| **Sex** (%) |  |  |  |
| Male | 2（50.0%） | 1（33.3%） | 2（66.7%） |
| Female | 2（50.0%） | 2（66.7%） | 1（33.3%） |
| **Occupation** |  |  |  |
| Worker | 1 (25.0%) | 1 (33.3%) | N/A |
| farmer | 0 (0.0%) | 2 (66.7%) | N/A |
| office clerk | 3 (75.0%) | 0 (0.0%) | N/A |
| **Education Background** (%) |  |  |  |
| Lack of education | 0 (0.0%) | 1(33.3%) | N/A |
| Below high school | 1 (25.0%) | 2 (66.7%) | N/A |
| High school and above | 3 (75.0%) | 0 (0.0%) | N/A |
| **RF** |  |  | N/A |
| Positive | 1 (25.0%) | 3 (100.0%) |  |
| Negative | 3 (75.0%) | 0 (0.0%) |  |
| **Anti-CCP** |  |  | N/A |
| Positive | 1 (25.0%) | 3 (100.0%) |  |
| Negative | 3 (75.0%) | 0 (0.0%) |  |
| **ESR** (SD) | 56.0 (9.54) | 93.67 (30.01) | N/A |
| **CRP** (IQR) | 24.67 (21.64) | 48.63 (52.88) | N/A |

**Table S2. The top 25 up-regulated and down-regulated differential miRNAs in PR patients compared RA patients and healthy individuals**

| **Up- regulated (log2 FC)** | | | **Down- regulated (log2 FC)** | | |
| --- | --- | --- | --- | --- | --- |
| **PR** | **vs Con** | **vs RA** | **PR** | **vs Con** | **vs RA** |
| hsa-miR-186-3p | 10.21 | 7.57 | hsa-miR-382-3p | -9.03 | -8.67 |
| hsa-miR-8061 | 7.16 | 7.20 | hsa-miR-329-5p | -7.22 | -7.56 |
| hsa-miR-3609 | 6.06 | 6.35 | hsa-miR-6515-5p | -6.64 | -5.94 |
| hsa-miR-3168 | 5.54 | 6.12 | hsa-miR-383-5p | -5.90 | -5.86 |
| hsa-miR-208a-3p | 4.38 | 6.03 | hsa-miR-758-3p | -5.82 | -5.76 |
| hsa-miR-19b-3p | 3.96 | 3.94 | hsa-miR-124-5p | -5.75 | -5.75 |
| hsa-miR-19a-3p | 3.53 | 3.94 | hsa-miR-377-5p | -5.72 | -5.64 |
| hsa-miR-33b-5p | 3.11 | 3.74 | hsa-miR-135a-5p | -5.58 | -5.39 |
| hsa-miR-144-5p | 2.87 | 3.71 | hsa-miR-206 | -5.56 | -5.33 |
| hsa-miR-20b-5p | 2.76 | 3.28 | hsa-miR-323b-3p | -5.49 | -5.28 |
| hsa-miR-106a-5p | 2.70 | 3.20 | hsa-miR-516b-5p | -5.45 | -5.09 |
| hsa-miR-181c-5p | 2.57 | 3.14 | hsa-miR-655-3p | -5.44 | -4.98 |
| hsa-miR-130a-3p | 2.51 | 3.07 | hsa-miR-379-3p | -5.42 | -4.94 |
| hsa-miR-548d-5p | 2.48 | 2.96 | hsa-miR-124-3p | -5.38 | -4.93 |
| hsa-miR-548ay-5p | 2.44 | 2.93 | hsa-miR-1298-5p | -5.26 | -4.80 |
| hsa-miR-200a-5p | 2.33 | 2.27 | hsa-miR-219a-2-3p | -5.17 | -4.69 |
| hsa-miR-33a-5p | 2.32 | 2.13 | hsa-miR-6529-5p | -5.14 | -4.68 |
| hsa-miR-552-3p | 2.28 | 2.05 | hsa-miR-369-3p | -4.94 | -4.67 |
| hsa-miR-5100 | 2.24 | 1.97 | hsa-miR-205-5p | -4.48 | -4.30 |
| hsa-miR-10a-3p | 2.15 | 1.90 | hsa-miR-9-3p | -4.06 | -3.79 |
| hsa-miR-15a-5p | 2.14 | 1.81 | hsa-miR-9-5p | -3.95 | -3.77 |
| hsa-miR-376a-3p | 1.91 | 1.79 | hsa-miR-944 | -3.80 | -3.60 |
| hsa-miR-142-3p | 1.83 | 1.76 | hsa-miR-411-3p | -3.61 | -3.60 |
| hsa-miR-548ad-5p | 1.83 | 1.73 | hsa-miR-381-3p | -3.61 | -3.54 |
| hsa-miR-10a-5p | 1.79 | 1.73 | hsa-miR-384 | -3.43 | -3.27 |

Table S3 The Power Analysis of Exploration and Validation Cohorts (Gpower 3.1)

| The exploration cohort | | | | |
| --- | --- | --- | --- | --- |
| Analysis group | Sample size | Effect size (Cohen's d) | Statistical power (1-β) | Significance level (α) |
| PR vs healthy individuals | 4 vs 3 | 1.0 | 19% | 0.05 |
| PR vs healthy individuals | 4 vs 3 | 2.0 | 58% | 0.05 |
| RA vs healthy individuals | 3 vs 3 | 1.0 | 16% | 0.05 |
| RA vs healthy individuals | 3 vs 3 | 2.0 | 50% | 0.05 |
| The validation cohort | | | | |
| PR vs healthy individuals | 27 vs 31 | 1.0 | 89% | 0.05 |
| RA vs healthy individuals | 30 vs 31 | 1.0 | 85% | 0.05 |


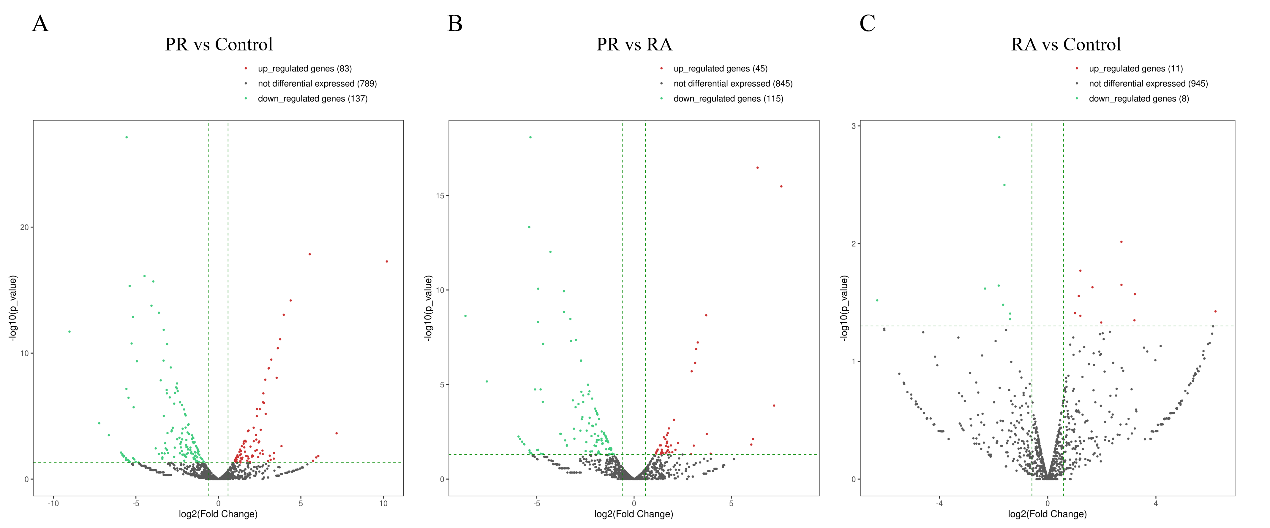


Figure S1. The volcano plots for the comparison between the groups.


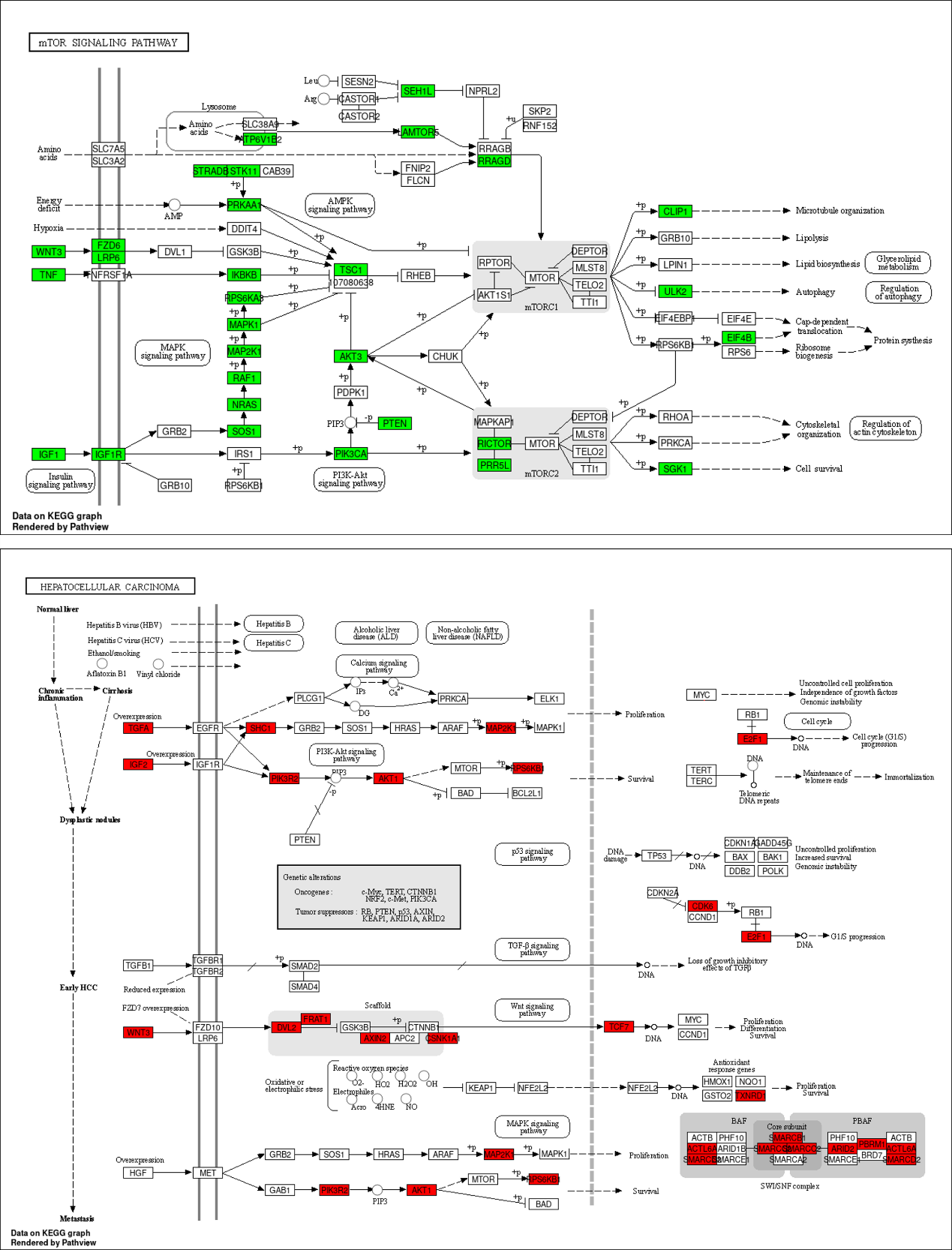


Figure S2. Signaling pathways differentially affected by miRNAs in mTOR signaling pathway and hepatocellular carcinoma pathway. Green marked nodes are associated with down-regulated genes, red marked nodes are associated with up-regulated or only whole dataset genes, white nodes have no significance.


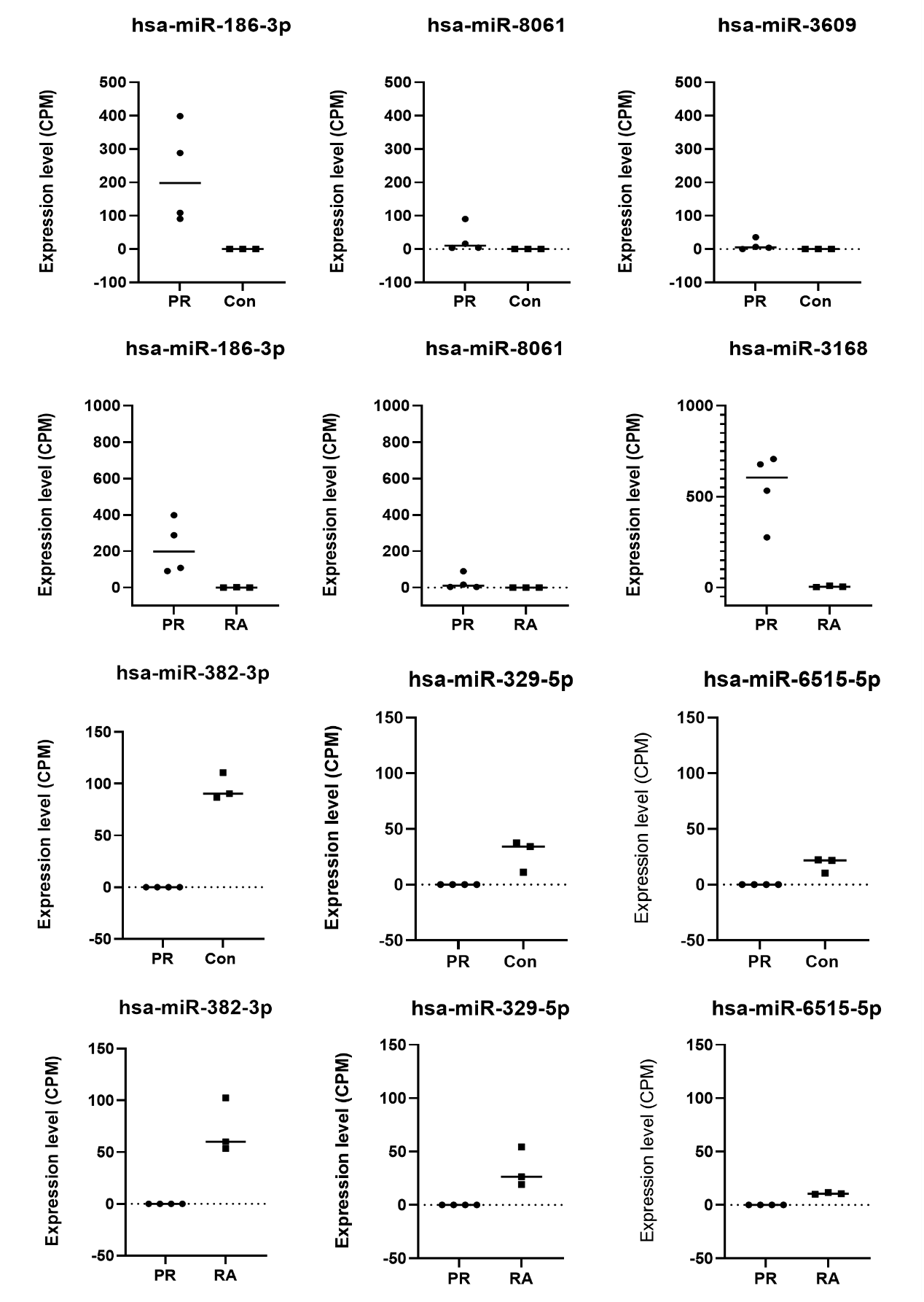


Figure S3. Among the up-regulated and down-regulated miRNAs, the top 3 miRNAs with the most significant expression differences in PR were compared RA patients and healthy individuals.


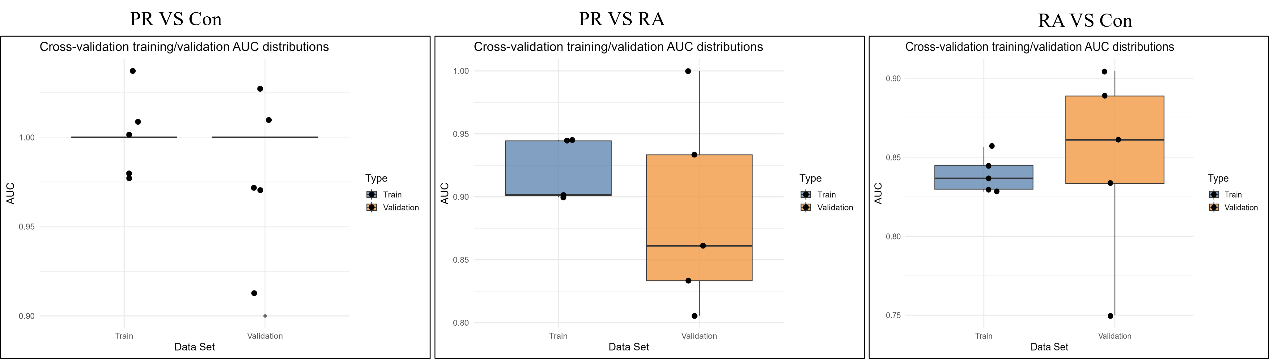


Figure S4. The AUC box plot for judging overfitting/underfitting
